# Congenital enteropathy caused by ezrin deficiency

**DOI:** 10.1007/s00439-025-02738-w

**Published:** 2025-03-26

**Authors:** Georg F. Vogel, Katharina M. C. Klee, Arzu Meltem Demir, Dorota Garczarczyk-Asim, Michael W. Hess, Lukas A. Huber, Thomas Müller, Andreas R. Janecke

**Affiliations:** 1 Department of Paediatrics I, Medical University of Innsbruck, Anichstrasse 35, Innsbruck, 6020 Austria; 2 Institute of Cell Biology, Biocenter, Medical University of Innsbruck, Innsbruck, 6020 Austria; 3 Ankara University, Faculty of Medicine, Department of Pediatrics, Division of Pediatric Gastroenterology, Hepatology and Nutrition, Ankara, 06110 Türkiye; 4 Institute of Histology und Embryology, Medical University of Innsbruck, Innsbruck, Austria; 5 Division of Human Genetics, Medical University of Innsbruck, Innsbruck, 6020 Austria

## Abstract

Ezrin, encoded by *EZR*, is a central module of epithelial polarity and links membrane proteins to the actin cytoskeleton directly or indirectly through scaffold proteins in the epithelium. Ezrin knockout mice fail to thrive and do not survive past weaning. We identified a homozygous *EZR* loss-of-function (LoF) variant, c.356dup, by exome sequencing in an infant with intractable diarrhea and failure to thrive, who died from septicemia at 5 months of age. The variant localized within a homozygous region of 13.2 Mb in the proband, is consistent with inheritance identical-by-descent from the consanguineous parents, and segregated with disease in the proband’s family. *EZR* transcript analyses in a heterozygous carrier showed that the variant triggers nonsense-mediated mRNA decay. Homozygous *EZR* LoF variants have not been reported in public databases. In this study, we generated a Caco-2 *EZR* knockout cell line to investigate the role of ezrin in human intestinal epithelia. Our analyses used electron and immunofluorescence microscopy to assess structural changes in the knockout cells. We observed significant disorganization of the terminal web region, microvillus rarefaction and abnormal branching. Furthermore, the absence of ezrin resulted in the mislocalization of the ezrin-interacting scaffold protein Na+/H + exchanger regulatory factor-1. In conclusion, this represents the first documentation of complete ezrin deficiency in humans, highlighting the essential and non-redundant functions of the protein in maintaining intestinal physiology.

## Introduction

Congenital diarrheas and enteropathies (CODEs) comprise a group of ultrarare diseases caused by variants in approximately 60 genes; variants disturb intestinal epithelial development and cell function (Babcock et al. 2023; Stallard et al. 2023), and in addition, a number of inborn errors of immunity (IEI) present with protracted diarrhea as the first or major sign in infancy (Uhlig et al., 2021). Infantile-onset, persistent diarrhea typically leads to a life-threatening situation with severe dehydration and serum electrolyte abnormalities. Infantile-onset secretory diarrhea and intestinal failure in CODEs require intricate interventions that frequently include long-term parenteral nutrition (PN). Secretory diarrhea can be distinguished from osmotic and functional diarrhea by virtue of higher stool volumes that continue despite fasting and occur at night while osmotic diarrhea results from luminal water retention due to poorly absorbed substances. Genetic testing, particularly exome sequencing (ES), has significantly changed the diagnostic landscape for CODEs, reducing the need for invasive procedures. The identification of pathogenic gene variants, accompanied by functional testing, has improved our understanding of these rare conditions (Babcock et al. 2023).

Proper apical and basolateral membrane polarization is fundamental for enterocyte function and intestinal homeostasis (Szabo et al. 2024). At the apical plasma membrane, ezrin, radixin, and moesin (ERM) proteins play a fundamental role in organizing cortical domains by assembling membrane protein complexes and linking them to the cortical actin cytoskeleton (McClatchey 2014). ERM proteins are characterized by a ∼300 amino acid, plasma membrane-associated FERM (four-point-one ezrin, radixin, moesin) domain, followed by a putative coiled-coil domain that contains a F-actin binding site (Turunen et al. 1994). ERM proteins exist as monomers, homo- or heterodimers, in which the tail domain is bound to the FERM domain. Upon binding of phosphatidylinositol 4,5-bisphosphate and phosphorylation of distinct threonine residues in the C-terminal domain, ERMs get activated, the C-terminal domain can bind to F-actin and the FERM domain becomes accessible for binding to specific interaction partners such as Na+/H + exchanger regulatory factor 1 (NHERF1) (Fehon et al. 2010). Both constitutive and conditional ezrin knockout (KO) mice fail to thrive postnatally, display increased stool volumes, and die at weaning (Casaletto et al. 2011; Saotome et al. 2004). Here, we report the first instance of a corresponding human phenotype, an infant with intractable diarrhea in whom a candidate disease-causing ezrin (*EZR*) variant, NM_001111077.2:c.356dup, was identified by ES. This variant was present in homozygous state, predicted to result in a frameshift and premature stop codon (NP_003370.2:p.Glu120*), and be subjected to nonsense-mediated mRNA decay (NMD).

## Materials and methods

### Patients

There is a diagnostic and research focus on inherited enteropathies at the Department of Paediatrics I, Medical University of Innsbruck. Patients and parents’ samples are referred continuously for genetic testing to identify the etiology of the disorder. Written informed consent for genetic testing and correlation with clinical data was obtained from the proband′s parents, and the studies were approved by the local ethics committee (vote no. AN2016-002, 10/2021). ES is routinely performed, and data evaluation first focuses on known CODEs (panel analyses) (Babcock et al. 2023). When no etiology is identified, the analysis is eventually extended to the whole ES set of genes in order to identify potentially causative variants.

### Exome sequencing, variant validation, and intrafamilial segregation analysis

**Fig. 1:**
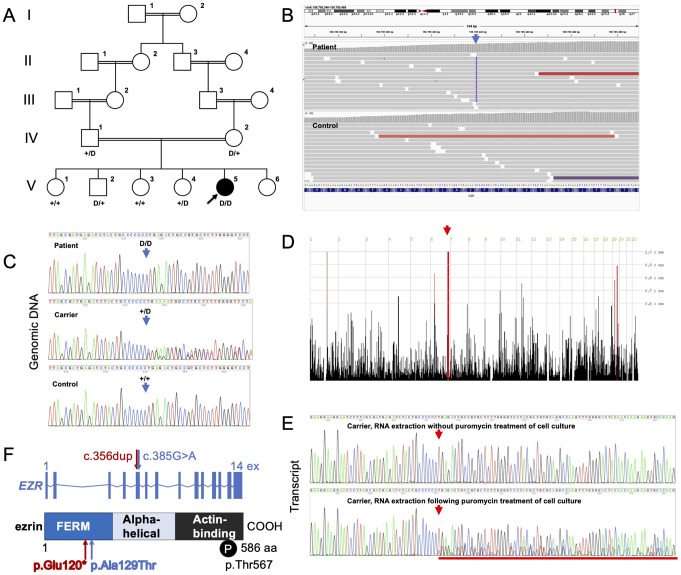
Simplified pedigree of the family under study and *EZR* 1-bp deletion (delineated by D) segregation (**A**), documentation of the *EZR* variant by ES (**B**) and Sanger sequencing (**C**) (arrows), and the localization of the *EZR* gene within a large region of homozygous variants (**D**). The EZR transcript harboring the NM_001111077.2:c.356dup variant undergoes NMD as demonstrated by Sanger sequencing in RNA derived from variant carrier fibroblasts untreated (upper panel) and treated (lower panel) with puromycin to inhibit NMD. A mixture of mutant and wild-type transcript is seen with NMD inhibition, underlined by a red bar (**E**). (**F**) Localization of the c.356dup and the reported c.385G > V variants in the ezrin gene and protein (with domains indicated); ex, exon; P, phosphorylation

Genomic DNA was isolated from peripheral leukocytes by standard procedures. DNA samples from the proband, her healthy parents, and four healthy siblings were available (Fig. 1). ES was performed with the proband’s DNA sample using Agilent’s Sureselect xt2 V6 enrichment kit, respectively, and an Illumina HiSeq4000 instrument, generating 100-bp paired-end reads that were aligned to the human reference genome GRCh37 with Burrows-Wheeler transformation algorithm (Li and Durbin 2009). Polymerase chain reaction (PCR) duplicates were removed with PICARD (http://picard.sourceforge.net) and single nucleotide substitutions, and small indels were called with SAMtools software. Sequencing reads were also aligned to the human reference genome GRCh38 and variants were called with the Genome Analysis Toolkit (GATK) version 4.0 (https://github.com/broadinstitute/gatk). Variants were annotated and filtered against public variant databases with Ensembl’s Variant Effect Predictor (https://www.ensembl.org/info/docs/tools/vep/index.html).

The resulting lists of sequence variants were filtered for an allele frequency below 0.005, and for a predicted effect on protein function (missense, stop gain, frameshift, inframe insertion and deletion, canonical splice-site alteration). Pathogenic or likely pathogenic variants were preferentially searched for in genes associated with those listed in the following gene panels obtained from Genomics England (https://panelapp.genomicsengland.co.uk/panels/) “Intestinal failure or congenital diarrhoea (Version 3.5),” “Primary immunodeficiency or monogenic inflammatory bowel disease (Version 7.21).” The lists were also filtered manually with respect to rare variants with an effect on protein function. All variants were evaluated in silico for pathogenicity using CADD (http://cadd.gs.washington.edu/score) (Rentzsch et al. 2019); missense variants were evaluated by PolyPhen-2 (http://genetics.bwh.harvard.edu/pph2) (Adzhubei et al. 2010) and SIFT (Ng and Henikoff 2003); and splice site variants were evaluated using SpliceAI lookup (https://spliceailookup.broadinstitute.org/) (Jaganathan et al. 2019). GRCh37 ES data were evaluated for copy-number variants (CNVs), including single exon deletions and duplications with the panelcn.MOPS v1.28.0 software (Povysil et al. 2017).

The current ‘‘gold standard’’ human genome reference assembly is GRCh37 in our diagnostic setting, where more than 40,000 exome data sets are available as controls, in addition to public databases. Aligning to both GRCh37 and GRCh38 reference sequences is currently performed for samples who remained negative for pathogenic or likely pathogenic variants in well-established disease genes following GRCh37 alignment. There appears to be a good agreement between both alignments and variant call datasets generally in our institute, but this has not been evaluated systematically yet. Exome variant discrepancies due to reference-genome differences and with respect to monogenic and multifactorial diseases were reported (Guo et al. 2017; Li et al. 2021; Vogel et al. 2024).

The *EZR* variant designation is based on the National center for biotechnology information reference transcript NM_001111077.2 (corresponding to Ensembl transcript reference sequence ENST00000367075.4) and the genomic reference sequence NG_052952.1 (corresponding to Ensembl gene ENSG00000092820). The exon numbering is based on NG_052952.1. Nucleotide numbering uses + 1 as the A of the ATG translation initiation codon in the reference sequence, with the initiation codon as codon 1. Sanger sequencing of a genomic, 561-bp PCR fragment containing *EZR* exon 5, produced with forward primer 5’-TGTGGTAGCACTTTTGACTAACC-3’ and reverse primer 5’-ACTGGCGAAGTCCTTGTGTT-3’ permitted *EZR* variant validation and segregation within the family.

### Primary fibroblasts culture, and EZR expression analysis

Primary fibroblasts from the proband’s father and control individuals were cultured to ∼90% confluency in Dulbecco´s Modified Eagle’s Medium (DMEM/F-12, Corning), supplemented with 100 U/ mL penicillin (Sigma-Aldrich), 100 µg/mL streptomycin (Sigma-Aldrich) and 10% FBS (Gibco) in a humidified atmosphere with 5% CO2 at 37°C. Cells were synchronized by serum starvation to enrich for mitotic phase cells. Cells were lysed with QIAzol solution (Qiagen) and total RNA was extracted, with and without puromycin addition to the medium (to a final concentration of 200 µg/ml) 6 hours before harvesting the cells, according to the manufacturer’s recommendations (RNeasy Plus Universal Mini kit, Qiagen). cDNA synthesis was done according to the protocol provided by the Thermo Fisher Scientific RevertAid First Strand cDNA synthesis kit. A 560-bp fragment of the *EZR* transcript (exons 3 to 7) was PCR-amplified from cDNA using the primer pair 5’-CTGGAGTTTGCAATCCAGCC-3’ and 5’-CCATACATTTCCAGGTCCTGAGC-3’ and directly sequenced.

### Cell lines, cell culture, cloning and genome editing

HEK293T and Caco-2 cells (ATCC) were cultured in DMEM (Sigma-Aldrich) containing high glucose, sodium pyruvate, 100 U/ mL penicillin (Sigma-Aldrich), 100 µg/mL streptomycin (Sigma-Aldrich) and 10% FBS (Gibco) in a humidified atmosphere with 5% CO2 at 37°C. *NHERF1* was amplified via PCR from cDNA and ligated into a pENTR-HA-Strep (a gift from S. Geley, Innsbruck Medical University, Innsbruck, Austria). Testing several antibodies to visualize endogenous NHERF1 expression by immunofluorescence microscopy was unsuccessful and we therefore resorted to ectopic expression and visualization via the HA-tag. For lentiviral transduction, cDNA was further subcloned via LR clonase into a pCCL-EF1α-BlastiR-DEST lentiviral vector using Gateway Cloning Technology (Invitrogen). For CRISPR/Cas9-mediated depletion, guide RNA (gRNA) targeting sequence for human *EZR* (5ʹ-CTGAGCGGCTGATCCCTCAA-3’) was selected using an online prediction tool [CHOPCHOP (Labun et al. 2016)]. The gRNA was cloned into a lentiCRISPRv2 vector via BsmBI restriction enzyme sites. lentiCRISPRv2 was a gift from F. Zhang [Massachusetts Institute of Technology, Cambridge, MA; Addgene plasmid 52961; (Sanjana et al. 2014)]. Lentiviral plasmids were cotransfected with Lipofectamine LTX (Invitrogen) together with pVSV-G and psPAX2 in the Hek293LTV producer cell line. Viral supernatant was harvested 48 and 72 h after transfection and directly used for CaCo2 cell infection. 6 d after infection, cells were selected with 10 µg/ml puromycin (Sigma-Aldrich) or 20 µg/ml blasticidin S (Invitrogen), as previously described (Vogel et al. 2015). Depletion efficiency was verified via Western blotting (WB), and a gRNA-resistant cDNA was used for rescue experiments. One clone of parental Caco-2 cells was used for subsequent experiments. Colonies of transduced cells were selected by limiting dilution. Cells were cultured, sub-cultured and used for Western blot experiments on 10 cm culture dishes (Sarsted, #83.3902). For experiments requiring fully polarized growth conditions [e.g., immunofluorescence (IF) experiments, transepithelial electrical resistance (TEER) and dextran permeability measurements], Caco-2 cells were seeded on 24-mm (Costar Transwell; pore size of 0.4 μm; Corning) and cultured for 14 days.

### Antibodies and reagents

Primary antibodies used in this study include those against ezrin (WB, 1:1000, 610602, Transduction Laboratories), GAPDH (WB, 1:1000, 97166 S, Cell Signaling) and HA (IF, 1:500, MMS-101R, Covance). Secondary horseradish-peroxidase-conjugated goat anti–mouse and goat anti-rabbit (1:5000; Sigma-Aldrich) antibodies were used for WB. Actin filaments were labeled with phalloidin–Alexa Fluor 568 (1:500; Life Technologies). Secondary Alexa Fluor-conjugated (Alexa Fluor 488 and 568) goat anti-mouse (1:1000; Life Technologies), goat anti -rabbit (1:1000; Life Technologies), and Hoechst 3342 (1:10,000; Thermo Fisher Scientific) were used for IF labeling.

### Western blotting

WB was performed as described previously (Fialka et al. 1997). Polyvinylidene fluoride membranes were incubated with primary antibody at room temperature (RT) for 1 h or overnight at 4 °C. Secondary antibody incubation was performed for 1 h at RT. Chemiluminescence was exposed on films.

### Immunofluorescence microscopy

Immunofluorescence labeling of Caco-2 cells grown on Transwell filters were performed as described previously (Vogel et al. 2015) and mounted in Mowiol. Samples were analyzed at RT with an epifluorescence microscope (Axio Imager M1; Carl Zeiss) equipped with a charge-coupled device camera (SPOT Xplorer; Visitron Systems) and recorded with VisiView 2.0.3 (Visitron Systems). Objective lenses used were a 25 × oil immersion objective (numerical aperture of 0.8) and a 10 × air objective (numerical aperture of 0.3; Carl Zeiss). Single confocal planes or stacks were recorded with a confocal fluorescence microscope (SP5; Leica) using a glycerol 63 × lens with a numerical aperture of 1.3 (Leica) at RT and mounted in Mowiol. The recording software used was LASAF 2.7.3 (Leica). Images were deconvolved with Huygens Professional Deconvolution and Analysis Software (Scientific Volume Imaging), and adjusted for brightness, contrast, and pixel size.

### Transmission electron microscopy

Transmission electron microscopy was performed as described previously (Ruemmele et al. 2010; Vogel et al. 2015a). In brief, 2D cultures were cryofixed, freeze substituted, and embedded in plastic (Ruemmele et al. 2010). Thin sections were recorded on a transmission electron microscope. Image processing was optionally carried out with iTEM-analySIS five software (from OSIS).

## Results

### Phenotype of EZR-related disease

The female proband was born to healthy parents, who are first cousins, at 37 weeks of gestation with a birth weight of 2330 g. Five siblings are healthy (Fig. 1A). Pregnancy was uneventful, there was no polyhydramnios. On examination, the proband had no dysmorphic features. Diarrhea started on postnatal day 3, accompanied by hypernatremic dehydration.

Initial stool analysis revealed a pH of 5 with positive reducing substances, raising the suspicion of carbohydrate malabsorption. A lactose-free formula was introduced, followed by a galactomin-19 formula, but the diarrhea persisted.

Immunoreactive trypsinogen was positive. She was transported to a tertiary center at age 35 days, dehydrated and weighing 1800 g. She had watery, sometimes bloody stools, with some improvement seen with total PN for three days. Introduction of amino-acid based formula (Neocate) led to watery, at times bloody stools 7–10 times/day. A representative blood gas analysis at the time read pH 7.45, HCO3 19.9 mEq/L (22–26 mEq/L), with stool electrolytes, Cl 72 mEq/L, Na 118 mEq/L, K 14 mEg/L, and an osmotic gap of 26 mOsm/kg. Serum sodium and chloride levels were normal with 2–3 mEq/kg sodium per day. Hypokalemia of 2.5 mEq/L was treated with 30 mEq/L in total PN. Stool pH was 5.5 at the time, and reducing substances were negative, and steatocrit was normal. The diarrhea ceased with total PN suggesting osmotic diarrhea, but the low stool osmotic gap suggested secretory diarrhea. Persistent hypoglycemia with normal serum insulin levels required glucose infusion of up to 12 mg/kg/minute. Thyroid function tests were normal. Her motor and cognitive development was appropriate for age. The patient experienced several episodes of septicemia, including sepsis with candida albicans, and neutropenia was noticed during these episodes. The patient died from septicemia at 5 months of age, weighing only 3300 g despite total PN for weeks. Intestinal biopsy material was not obtained; autopsy was not performed.

### Identification of *EZR* as a CODEs candidate

ES identified a homozygous *EZR* variant NM_003379.4:c.356dup (p.Glu120*) in the proband (Fig. 1B, C). Inspection of her ES data showed that the *EZR* variant localized within a 13.2-Mb region of homozygosity in the proband indicating inheritance identical-by-descent from the consanguineous parents (Fig. 1D), and supporting autosomal recessive inheritance of the proband’s disorder. Other potentially pathogenic rare or private variants that might explain the phenotype were neither seen within coding regions or at splice sites in genes known to cause CODEs or IEI, nor in other genes covered by ES. Follow-up Sanger sequencing demonstrated segregation of the *EZR* variant with disease (Fig. 1A). This *EZR* variant (rs923993817) is present in the Genome Aggregation Database (gnomAD; https://gnomad.broadinstitute.org/) in the heterozygous state only, with a global minor allele frequency of 0.00001425, consistent with the low prevalence of the CODEs spectrum in the general population. The LOEUF metric from gnomAD shows a trend towards intolerance of *EZR* haploinsufficiency (gnomAD v2.1.1 LOEUF score: 0.44, pLI score: 0.18; gnomAD v4.1.0 LOEUF score: 0.7, pLI score: 0), and homozygous *EZR* LoF variants are not found in gnomAD. The variant identified in the proband localizes to *EZR* exon 5 of 14, and is thus expected to trigger NMD of the mutant transcript. This was indeed demonstrated in a carrier of the c.356dup variant: cDNA was synthesized both with RNA derived from fibroblasts that were treated with puromycin to inhibit the NMD machinery prior to cell harvesting, and with RNA from untreated fibroblasts. Using EZR transcript-specific primers and PCR conditions, Sanger sequencing detected only the wild-type transcript in fibroblasts with intact NMD machinery (Fig. 1E, upper panel) but detected an additional aberrant transcript containing the c.356dup variant when the NMD machinery was inhibited. This aberrant transcript was present at a lower extent when compared to the wild-type transcript (Fig. 1E, lower panel). As this result demonstrated the loss of the mutant transcript, we did not perform Western blotting for EZR protein expression in the carrier’s fibroblasts compared with controls. Collectively, our data indicate pathogenicity and LoF of the *EZR* c.356dup variant.

### Ezrin is required for the integrity of the terminal web and the brush border in human colon carcinoma cell line Caco-2

**Fig. 2:**
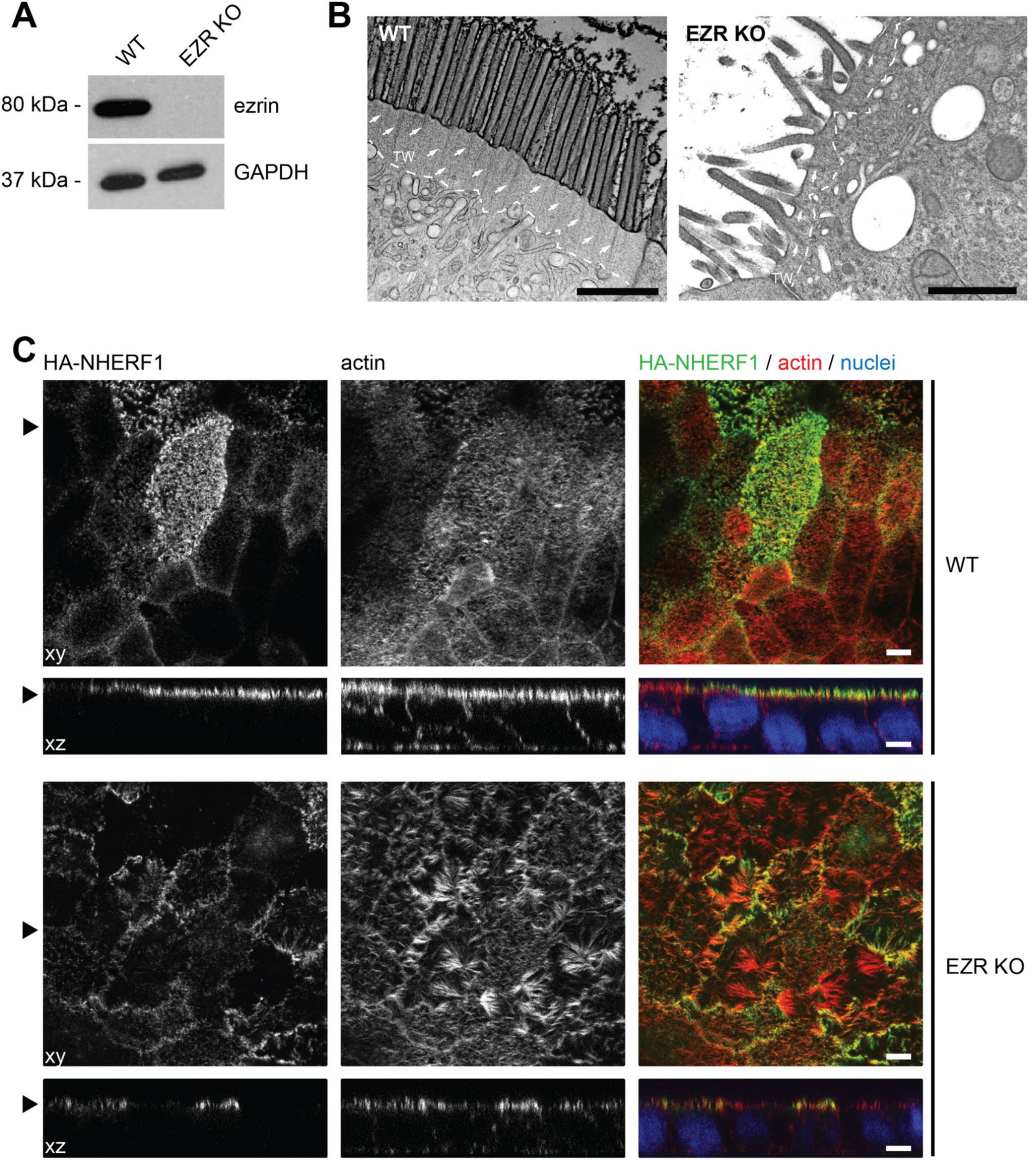
(**A**) Western blot confirming depleting of ezrin protein levels in *EZR* knockout (KO) Caco-2 cells. GAPDH is shown as loading control. (**B**) Electron micrographs of the apical brush region of wild-type (WT) and *EZR* KO Caco-2 cells. The terminal web (TW) is marked by the white dashed line and actin rootlets are indicated by white arrows. Scale = 1 μm. (**C**) Confocal IF micrographs of Caco-2 WT and *EZR* KO monolayers. NHERF1 (green) shows disturbed apical localization and microvilli and apical brush border actin (red) are aberrantly in *EZR* KO cells. XY = top view of polarized monolayer; XZ/YZ = lateral view of polarized monolayer. Scale = 5 μm

To investigate the effects of ezrin loss on enterocyte function, we generated a CRISPR-Cas9-mediated ezrin KO in the Caco-2 intestinal epithelial cell line, which was subsequently validated by Western blot analysis (Fig. 2A). Transmission electron microscopy of cryosectioned polarized Caco-2 cells revealed a pronounced rarefaction and deformation of the apical microvilli, together with a marked reduction in the actin filament network of the subapical terminal web (Fig. 2B). In addition, ezrin depletion resulted in aberrant and reduced apical localization of its interacting protein ectopically expressed NHERF1 (Fig. 2C), highlighting the critical role of ezrin in maintaining structural integrity and protein localization within the intestinal epithelium.

## Discussion

We identified a homozygous *EZR* null variant in an infant with intractable diarrhea of congenital onset and with complete failure to thrive. Evidence for pathogenicity of this ultrarare variant is provided by variant segregation analysis within the proband’s family, inheritance of the variant identical-by-descent, and in that homozygous LoF variants are not found in the gnomAD population database despite adequate coverage of this gene. Further, ES in the patient did not disclose any other pathogenic or likely pathogenic variant in any of the genes known to cause CODEs or IEI that would explain the severe diarrhea in this patient. As ezrin is highly expressed in human intestinal epithelium and plays roles in cell surface structure adhesion, migration and organization, the identified *EZR* LoF variant was considered an excellent candidate to explain the diarrhea in our patient by affecting the establishment and the maintenance of the apical plasma membrane structure and composition.

Previously, an isolated case of B-cell deficiency and impaired adaptive immune function was reported in association with a homozygous p.Ala129Thr variant (rs528409234). At the time of reporting, the affected individual was 30 years old with normal stature and without gastrointestinal symptoms. The mutant ezrin was found to be expressed in peripheral blood mononuclear cells at slightly lower levels compared to controls. Phosphorylation at tyrosine-146 and tyrosine-353 residues was absent (Garcia-Solis et al. 2023), however, how these modifications affect the conformation and function of ezrin is unclear, and how the mutant ezrin produced the immunological phenotype in their patient remained unsolved. The markedly different phenotypes observed in this individual and our proband may be due to residual ezrin function conferred by the p.Ala129Thr variant in the intestinal tract, as well as the influence of genetic and non-genetic modifiers. Furthermore, as the *EZR* variants were each identified through ES in single individuals who were born to consanguineous parents, this leaves the possibility that disease-related intronic, regulatory or structural variants at other gene loci might have been missed. Of note, B cell development, as well as the proportion and numbers of major B cell subsets in peripheral lymphoid organs, were unaffected by the loss of ezrin in conditional ezrin knockout mice (Pore et al. 2013), and despite high expression in lymphoid precursor cells, ezrin was dispensable for lymphoid development, likely due to redundancy with moesin (Shaffer et al. 2010).

Our clinical and cell biological observations lead us to conclude that ezrin deficiency may be associated with severe enteropathy in humans, of congenital onset as in the patient we report here.

It is tempting to speculate that ezrin deficiency might lead to fetal demise given that biallelic LoF variants are not represented in the gnomAD database. The idea of an early-onset, severe enteropathy is strongly supported by both constitutional and conditional ezrin deletion studies in mice. Constitutional deletion of ezrin resulted in severe defects in the apical integrity of the entire developing intestinal epithelium and ultimately in neonatal mortality, underlining the critical role of ezrin in intestinal development and function (Saotome et al. 2004). Requirement for ezrin in the intestinal epithelium was confirmed by producing this same phenotype in mice with an ezrin deletion confined to the intestinal epithelium (Casaletto et al. 2011). Tamoxifen-induced ezrin deletion in adult mice bypassed the neonatal lethality but resulted in severe defects in apical integrity that were nearly identical to that seen in constitutional and conditional neonates in which ezrin was deleted before villus morphogenesis (Casaletto et al. 2011). Mouse intestinal epithelial cells showed rarefied and deformed apical microvilli, a phenotype similar to the one observed in *EZR* KO Caco-2 cells. However, while mouse *Ezr* null enterocytes showed an aberrantly thickened and disorganized terminal web, *EZR* depletion resulted in a less prominent terminal web in Caco-2 cells. The intestinal epithelium-derived, polarized Caco-2 cells have been used earlier to model enteropathies such as microvillus inclusion disease (MVID), a genetically heterogenous type of congenital secretory diarrhea (Aldrian et al. 2021; Janecke et al. 2021; Vogel et al. 2017b). The severe clinical symptoms in MVID and ezrin deficiency arise from a severe reduction in the absorptive capacity of the intestine due to a combination of villus and microvillus defects.

The defects at the apical brush border seen in ezrin deficient Caco-2 cells, and in the intestine of ezrin KO mice share similarities with those seen in MVID. However, a number of differences also exist between the small intestine in ezrin KO mice and MVID patients. Brush border enzymes alkaline phosphatase and sucrase isomaltase accumulated intracellularly in MVID enterocytes (Cutz et al. 1989; Phillips et al. 1985, 2004), but remained at the apical surface of intestinal epithelial cells in ezrin KO mice (Casaletto et al. 2011; Saotome et al. 2004). Characteristic findings of MVID, such as ectopic microvilli forming microvillus inclusions of variable morphology and basolateral subapical accumulations of pleomorphic vesicles - including numerous PAS-positive “secretory granules” - were not observed in the enterocytes of ezrin KO mice or in EZR KO Caco-2 cells. While the loss of apical ezrin, or possibly its phosphorylation at threonine-567, may explain the abnormalities in the brush border and terminal web, it does not explain the intracellular retention of brush border proteins and the formation of microvillus inclusions seen in MVID enterocytes.

Ezrin has been identified as an apical marker in intestinal epithelial cells that was lost from the apical membrane and accumulated either in the cytoplasm or on the basal side of enterocytes in MVID patients, contributing to the conclusion of MVID representing a cell polarity defect (Ameen and Salas 2000; Michaux et al. 2016). In addition, the actin-organizing scaffold protein ezrin was shown to contribute to microvilli development and organization at the apical surface of epithelial cells (Fehon et al. 2010). Interestingly, ezrin LoF was suggested to represent a downstream consequence of myosin VB LoF and part of MVID pathogenesis. This suggestion was based on the observation that myosin VB LoF in intestinal epithelial cell lines, as well as in patients carrying myosin VB variants, caused an aberrant localization of Rab11a-positive recycling endosomes with a concomitant inhibition of ezrin phosphorylation and of microvilli development (Dhekne et al. 2014).

Ezrin and NHERF1 function are crucial for apical localization of Na+/H + exchanger 3 (NHE3) (Zhao et al. 2004). Direct binding of NHE3 to ezrin is necessary for basal exocytosis, delivery from the synthetic pathway and movement of NHE3 in the brush border; NHE3 is regulated by binding indirectly to ezrin via NHERF1 (Cha and Donowitz 2008). Ezrin depletion in Caco-2 cells led to a mislocalization of HA-tagged NHERF1. Presumably, this would result in mistargeting and impaired NHE3 function in patients, a pattern that is also seen in MVID patients (Vogel et al. 2017; Vogel, Klee, Vogel et al. 2015a, b; Vogel, van Rijn, Vogel et al. 2017a, b). Impaired NHE3 function is considered a driver of diarrhea (Priyamvada et al. 2015), and NHE3 deficiency caused both by variants in its encoding gene and through downregulation by increased intracellular cyclic guanosine monophosphate results in a secretory diarrhea as well (Janecke et al. 2015; Muller et al. 2016).

A limitation of our study is the fact that we were unable to identify further patients with ezrin deficiency resulting from biallelic LoF variants in *EZR*, as well as the unavailability of proper antibodies against NHERF1 and NHE3, which impacted the in vitro functional studies.

Taken together, however, our findings provide compelling evidence that the homozygous *EZR* LoF variant, c.356dup, underlies the severe enteropathy observed in the patient described here.

## Data Availability

No datasets were generated or analysed during the current study.
